# Influence of Filler Particle Sizes on the Physical Properties of Bulk-Fill Composites Compared to Conventional Composites

**DOI:** 10.7759/cureus.36032

**Published:** 2023-03-11

**Authors:** Apurva Nagrale, Siddhi Nevrekar, Sahil Kawle, Harshraj Gawande, Jaiti Gupte, Shashank Gaikwad

**Affiliations:** 1 Department of Conservative Dentistry & Endodontics, Dr. G.D. Pol Foundation's YMT Dental College and Hospital, Navi Mumbai, IND; 2 Department of Orthodontics, Bharati Vidyapeeth Dental College and Hospital, Navi Mumbai, IND

**Keywords:** nanocomposites, composites, filler particles, flexural strength, tensile strength, compressive strength

## Abstract

Introduction: Filler size affects how the material is coated and finished, while filler stacking affects how strong the material is, how flexible it is, how resistant it is to wear, and how much it shrinks when it polymerizes. The purpose of this research was to compare micro-hybrid, nano-composite, and bulk-fill composites with respect to their compressive strength, diametric tensile strength, and flexural strength.

Materials and methods: To organize the samples according to International Organization for Standardization (ISO) 4049 and American Dental Association (ADA) detail number 27, we used a custom-made Teflon mold. A total of 45 samples were used, with 15 samples in each group. The sample was mounted on a state-of-the-art general testing machine to determine its compressive strength and polar rigidity. The 3-point bowing test was used to determine flexural strength. A one-way analysis of variance (ANOVA) was used for quantitative analysis, followed by a post hoc test with a significance level of P < 0.05.

Results: The Tetric N Ceram Bulk Fill, the Filtek Z350 XT nanocomposite, and the T-Econom micro-hybrid composite all had different levels of flexural and compressive strength. This difference was statistically significant. Nanocomposites have superior compressive and flexural strengths to their counterparts analyzed in the present study.

Conclusion: Nano-composite combines the properties of being strong and looking good. It can be used in both front and back restorations that need to be strong enough to withstand the forces of chewing.

## Introduction

After composite materials were used in dentistry for the first time in the 1960s, unsightly amalgam fillings in the front teeth were quickly replaced. In the 1990s, composites took the place of amalgam as the most common filling material [1]. This was the beginning of a new era of non-invasive dentistry. This steady upswing was possible owing to the contemporaneous advancement in adhesive dentistry [2]. Resin composites have evolved through generations based on filler composition, such as traditional (macro-filled) composites, micro-filled composites, hybrid composites, micro-hybrid composites, and nanocomposites [3]. The mechanical qualities of the recycled material may be altered by the use of fillers. The polishability and renovation of the reclamation are affected by filler size, while filler stacking influences the material's strength, elastic modulus, wear obstruction, and polymerization shrinkage [4-7]. In the primary composites, only full-scale fillers were used. As expected, the shrinkage conductivity and flexural modulus of these fully filled composites were found to be in the positive range. Wear resistance was poor, and it was regrettable that their surface properties were inadequate. Miniature-filled composites made a splash since they were the first materials that could withstand wear well while maintaining a high-quality oral surface. However, despite its potential, miniature filler was unable to overcome two major obstacles: There is a lack of flexural strength and flexural modulus because, first, the composite cannot be built up as effectively as it might with large-scale fillers. Second, the high, unambiguous surface area of micro-fillers means that only small amounts may be used to significantly increase the composite's uniformity [8,9]. As a result, tiny, filled composites experienced significant shrinkage throughout the polymerization process. When it comes to improving composites, hybrid composites are the next logical step. For this reason, a large filler load was taken into account, which resulted in both increased real strength and reduced shrinkage during polymerization [9]. As a natural next step in filler innovation, the incorporation of nanometrically quantified filler particles in hybrid composite and the introduction of purely nano-filled composites are both welcome. Small in size and spherical in form, these filler particles expose a large surface area. While nano-half-and-half uses the method of combining nanometric and conventional filler particles, nano-filled composites only have nano-sized particles dispersed throughout the gum lattice. Nano-filled composites have better mechanical properties than micro-filled traditional composites. Thus, they come with burnishing properties equivalent to those of micro-filled composites and wear resistance on par with hybrid composites [10].

Photopolymerizable composite resin requires certain prerequisites, such as proper isolation, multiple methodical layering, and adequate curing time. In spite of their aesthetics and strength, composite restorations are time-consuming because of their unique incremental pattern of curing [11]. Bulk-fill resin-based composites (RBCs) reduce the need for this time-consuming method. They enable restorations to be cured in 4 mm-thick layers, thus minimizing the time required and resulting in better patient compliance [12]. Despite this quality, however, there has been a dearth of research evaluating the physical and mechanical aspects of bulk-fill RBC repairs. It is hypothesized that bulk-fill composites will have altered mechanical properties owing to their filler amount and particle size. Therefore, compressive strength, diametral elasticity, and flexural strength were among the mechanical parameters that the ebb and flow study aimed to estimate for micro-hybrid composites, nanocomposites, and mass-fill composites. Experts hypothesized that the filler size and filler stacking wouldn't have any effect on the evaluated composites' mechanical properties.

## Materials and methods

In order to evaluate the percentage of success in each group, the pilot study's findings were used to calculate an adequate sample size. The study was performed on cylindrical and cuboidal samples with 15 samples in each group under each testing category thus bringing the total sample size to 45 in each testing category. The groups were divided based on the following:

Group A: A Composite Material Used in Large Volumes (Tetric N Ceram Bulk Fill by Ivoclar Vivadent, Liechtenstein)

Group B: Nanocomposite (Filtek TM Z350XT, 3M ESPE, USA)

Group C: Microhybrid (Te-Econom, Ivoclair Vivadent, Schaan, Liechtenstein)

The samples were manufactured using a custom-built Teflon mold. Samples were manufactured for compressive strength testing having an interior diameter of 4 mm and a height of 6 mm. A total of 25mm length, 2 mm height, and 2mm width were prepared for flexural strength, according to International Organization for Standardization (ISO) 4049 standards. In order to evaluate polar stiffness, tests with a 6 mm internal width and a 3 mm level were prepared, as specified by American Dental Association (ADA) detail no. 27. After placing the form over the mylar strip (Dentsply Caulk), we next placed it over the glass shard. A 1 mm thick glass slide was placed on top of the composite and lightly compressed to create a flat, homogeneous surface. Composite on both sides was covered with mylar strips to ensure a smooth surface and to avoid oxygen-inhibited areas. For bulk fill composite, the core was built up in two layers, one of 4 mm and the other of 2 mm thickness. The Nanocomposites and Micro-hybrid composite were used incrementally, each increment of 2 mm thickness. With the help of ADA detail no. 27, tests were created with an internal width of 6 mm and a level of 3 mm to measure polar stiffness. We first adhered a piece of mylar (Dentsply Caulk) to the glass shard, and then we put the form on top of it. For a uniform and smooth surface, we put a glass slide with a thickness of 1 mm on top of the composite and softly squeezed it.

For compressive strength and diametral tensile strength

The evaluation of the properties was done by mounting the specimen on the custom-made jigs on a computerized Universal Testing Machine (Tinius Olsen H5KS, Redhill, United Kingdom) at a crosshead speed of 0.75±0.30 mm/min for compressive strength (CS) and 1 mm/min for diametric tensile strength (DTS). In a meticulously choreographed routine, each specimen was first placed upright for CS testing and then positioned uniformly for diametral elasticity testing at the base of the testing machine. They pressed down so hard on me that I was crushed. The average values of CS and DTS (in MPa) were calculated for each composite gum.

For flexural strength

Samples were bent at three different points to measure their flexural strength (FS), as specified by ISO 4049. The composites were applied to a Teflon mold, then molded between two parallel glass plates, coated with clear matrix strips, and then subjected to light curing to generate bar-shaped specimens (25 mm in length, 2 mm in width, and 2 mm in height). After being taken out of the mold, the specimens had their surfaces smoothed off so that there were no sharp corners or bumps. The samples were then placed on a stainless-steel support placed 20 mm apart. Universal Testing Machine was set at a crosshead speed of 0.75±0.30 mm/min for FS. The vertical load was exerted gradually, concentrating in the center until fracture. The values were recorded in MPa.

Statistical analysis

Data from Statistical Product and Service Solutions (SPSS) (IBM SPSS Statistics for Windows, Version 16.0, Chicago) software's FactSet Data Analysis Subset were dissected. The correlation between the groups was analyzed using an analysis of variance (ANOVA) test. In a series of Post Hoc Tuckey's Test comparisons, we looked at how Filtek Z350XT, Tetric N Ceram Mass fill, and T-Econom fared against one another in terms of CS, radial rigidity, and FS. This study compared two different composites simultaneously. With a P value less than 0.05, the results were determined to be statistically significant.

## Results

The present study aimed to access and compare the CS, DTS, and FS of three different composites i.e., Tetric N Ceram Bulk Fill (bulk-fill), Filtek Z350XT (Nanohybrid), and Te-Econom (Micro-hybrid) in megapascal (Table 1).

**Table 1:** Assessment of different composite based on their flexural, tensile, and compressive strength. TECO: Te-Econom composite BF: bulk fill composite Z350: Filtek Z350 composite

		N	Mean	Std. Deviation	Std. Error	95% Confidence Interval for Mean		Minimum	Maximum
						Lower Bound	Upper Bound		
Flexural strength in MPa	TECO	15	106.85	16.081	4.152	97.95	115.76	78	136
	BF	15	103.10	20.278	5.236	91.87	114.33	68	130
	Z350	15	146.92	6.965	1.798	143.07	150.78	138	159
	Total	45	118.96	25.117	3.744	111.41	126.50	68	159
Tensile strength in MPa	TECO	15	104.73	1.890	.488	103.68	105.77	102	109
	BF	15	108.22	1.637	.423	107.32	109.13	105	110
	Z350	15	125.54	1.118	.289	124.92	126.16	123	127
	Total	45	112.83	9.332	1.391	110.03	115.63	102	127
Compressive strength in MPa	TECO	15	162.25	2.492	.643	160.87	163.63	159	166
	BF	15	173.40	14.223	3.672	165.52	181.28	147	195
	Z350	15	277.28	2.487	.642	275.90	278.66	274	282
	Total	45	204.31	53.029	7.905	188.38	220.24	147	282

Compressive strength

**Figure 1:**
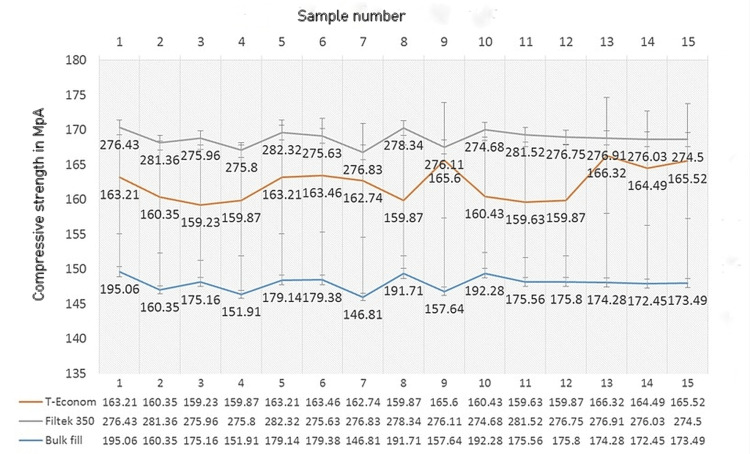
Comparison of compressive strength

ANOVA and Post Hoc Tukey’s test showed significant differences in the CS of all three composites irrespective of the pattern of curing. Filtek Z350XT emerged to be the strongest, followed by Tetric N Ceram Bulk Fill. The lowest strength was obtained for Te-Econom (Figure 1).

Diametral tensile strength

**Figure 2:**
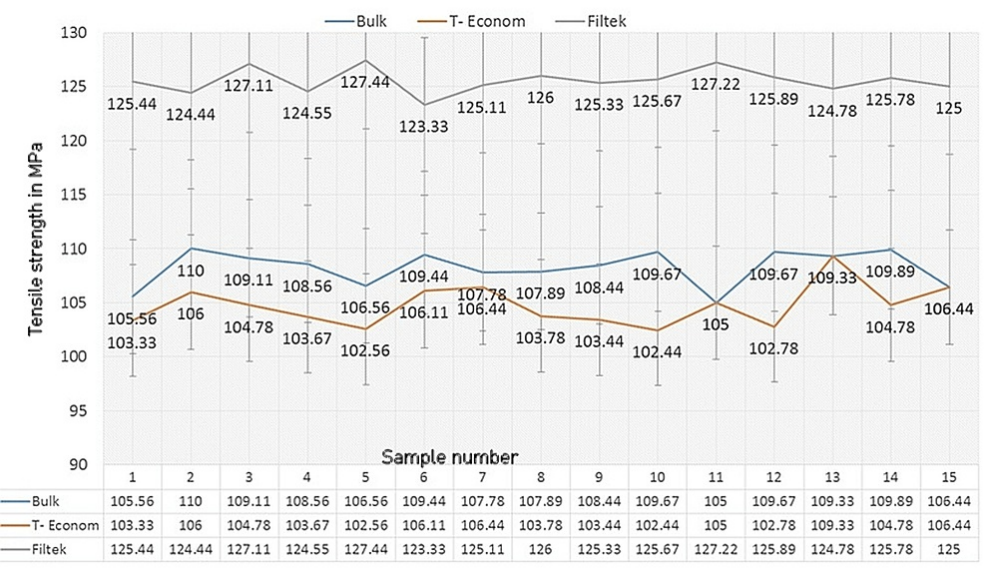
Comparison of tensile strength

Similarly, for the DTS, Filtek Z350XT proved to be superior with the highest value being 124.44 MPa, followed by Tetric N Ceram Bulk Fill and Te-Econom in descending order with values of 110.00 MPa and 109.33 MPa, respectively (Figure 2).

Flexural strength

**Figure 3:**
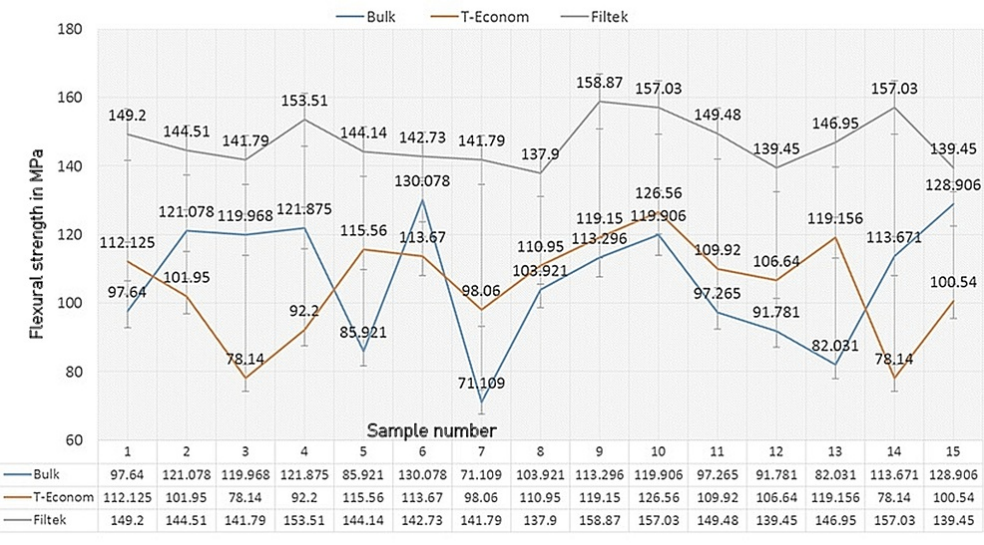
Comparison of flexural strength

In terms of FS, Nanocomposites Filtek Z350 XT made the top mark (158.87 MPa) followed by Tetric N Ceram Bulk Fill (130.078 MPa) and least by T-Econom (126.56 MPa) micro-hybrid composite. All the values are plotted in Table 2 along with the mean, standard deviation, and standard error (Figure 3).

Post Hoc Tukey’s Test revealed significant differences between all the pairs of each testing category except for the FS between Tetric N Ceram Bulk Fill and T-Econom micro-hybrid composite where the difference in the FS was not statistically significant; P value > 0.05 (Table 1).

## Discussion

Monomers and fillers are the core ingredients of light-curing filling materials. Additionally, they have monomer breakdown initiators, monomer breakdown stabilizers, and monomer breakdown-adding compounds. Tar composites' qualities have been rationally improved throughout time, and dental glues' adhesive strength has been simplified. Intriguingly, the photo-starters did not change for quite some time. However, recent developments have now been observed and achieved in this regard with the development of Ivocerin, a photo-initiator patented by Ivoclar Vivadent. In this study, the new technology nanocomposites Filtek Supreme 350XT had compressive, diametric tensile, and flexural strengths which were significantly higher than micro-hybrid and bulk fill composite. These results are consistent with those found in previous studies that evaluated the presentation of nanocomposites [13-15]. The nanocomposite Filtek Z350 XT is made up of both aggregated and nonaggregated nano silica fillers of 20 nm in size, as well as loosely bonded agglomerated zirconia/silica nano bunches with particle sizes ranging from 5 to 20 nm. Filler stacking is 78.5% by weight, and the range of group molecule sizes is 0.6-1.4 microns [16].

The addition of nanometer-sized particles to nanocluster designs lessens the interstitial dispersion of the filler particles. Furthermore, using spheroidal nanocluster fillers, which have a large molecular circulation, allows for greater filler stacking and superior real characteristics, much as the crossover composites [13,17]. One possible explanation for its superior presentation [18] is the increased contact surface of nanofillers with the natural stage in the nanocomposite. In the voids between the clusters, the nanometric particles find a home. The resulting surface is heavily laden with fillers due to this method. When nanocomposites are worn, only the vital particles in the nanocluster are severed, while the larger, less important particles are removed from the pitch. Thus the surface defects are smaller and the gloss retention is higher [19]. The pressure shift between particles is enhanced by increasing the filler stack height (particulate support). This reduces pressure on the pitch grid by redistributing occlusal tension from one molecule to the next. Since most composite saps are rather soft, refined particle reinforcement significantly increases the composites' pressure-bearing capabilities and provides a hardening effect.

The compressive, flexural, and diametric elasticity of a Tetric N Ceram mass fill composite was the second highest among all of the materials tested. The bulk fill composite contains, Ivocerin which is a germanium-based light-initiator that allows an increased depth of cure up to 4 mm. However, it also negatively impacts the mechanical property of bulk-fill composites. The increased depth of cure is further regulated by improving the translucency of the material. One strategy for doing this is cutting down on the amount of filler used. When the filler particles' dimensions are increased, the filler-matrix interface is reduced, reducing the scattering of light at the interface and allowing greater penetration of light into the material [20,21]. The change in the distribution and size of these filler particles could be the reason for the inferior performance of bulk fill composite in comparison to nanocomposite which has densely packed nanofillers.

The least mechanical properties were demonstrated by Te-Econom and Ivoclair Vivadent. Microfine composites have the lowest filler weight %. It has been studied in the literature that it is not only the percentage of filler but also the morphological characteristics of the filler particle that influence the mechanical property of the composite since it determines the filler loading. Filler particles that are spherical in shape have improved packing of the particles and hence higher resistance to fracture. They allow a more uniform distribution of stress rather than irregularly shaped particles that allow stress concentration [22]. Hence, the nanocomposite with more evenly distributed spherical-shaped particles had better mechanical properties than its counterparts.

CS, defined as the maximal stress a rigid material can bear when compressed along its length, is often utilized as an indicator of mastication resistance [23]. Since theoretically, a material may fail only by the separation of atomic planes (i.e., tensile failure), or by the slippage of atomic planes (i.e., compressive failure), this characteristic is meaningless (i.e., shear failure). While this is a drawback, a material's mechanical integrity may be gauged by whether or not its CS remains unchanged after extensive aging. For dental materials breaking brittlely, direct measurement of tensile strength is the most therapeutically relevant option [23]. Due to the tensile strains incurred while chewing, composites should break. Because of this, it is possible that the tensile strength of these materials is more clinically relevant than the CS [24]. The findings may not be reliable if the specimen deforms sufficiently before failure, which is a limitation of the test. In this investigation, the specimens showed little deformation before failure, suggesting that the diametral tensile test is a reliable indicator of the relative brittleness of the materials. One alternate method of gauging brittleness is to consider the material's FS in the transverse mode. Here, we used a three-point loading test as our methodology of choice. It's possible to use it as a paradigm for a clinical scenario brought on by the pressure of the obstructing cusp [25,26]. Classes I, II, III, and IV restorations, which are subject to the most force during mastication, need the use of materials with high FS and modulus to prevent catastrophic failures and preserve the marginal seal between the composites and the tooth substance. Features such as high quantum efficiency, high absorption capacity, and excellent bleaching qualities distinguish it. Ivocerin has enabled the development of new composite material, bulk fill material from Ivoclar Vivadent called Tetric N Ceram that can be cured in increments of 4 mm [27,28]. Bulk-fill composites and traditional composites are dissimilar in terms of filler loading and resin matrix composition. This means that the mechanical characteristics of these substances will likely differ. Since fracturing of composite resin restorations has been a key determinant of clinical failure [29], it is necessary to evaluate their physical behavior and the variables impacting the same in the laboratory if we are to properly anticipate the clinical results of direct restorations.

## Conclusions

Nanocomposites, due to the proportion and shape of the filler particles, have greater strength than their counterparts investigated in the present investigation, within the bounds of the current study. Nanocomposite combines the strength and aesthetic qualities needed for both front and back teeth restorations to resist masticatory pressures. However, since nanocomposite is a relatively new technology, more well-designed randomized clinical studies are needed to establish its long-term in vivo effectiveness. Polymerization shrinkage, conversion degree, and composite microleakage are a few other things that need to be looked at.
